# P-652. Evaluation of Empiric Antibiotic Use for Community Acquired Pneumonia

**DOI:** 10.1093/ofid/ofae631.849

**Published:** 2025-01-29

**Authors:** Kamryn Plechot, Helen Lee, Steven Park

**Affiliations:** UC Irvine Health, Orange, California; UCI Medical Center, Orange, California; University of California, Irvine, Irvine, California

## Abstract

**Background:**

Pneumonia is one of the most common infections resulting in hospitalization. Timely and appropriate empiric antibiotic treatment is crucial to improve prognosis in patients with community-acquired pneumonia (CAP). However, the use of empiric broad spectrum antibiotics is associated with serious consequences including development of antibiotic resistance and unintended adverse drug reactions. The Infectious Diseases Society of America (IDSA) guideline for the treatment of CAP recommends evaluation of specific risk factors to determine the need for empiric broad spectrum antibiotic coverage. The purpose of this study is to evaluate empiric antibiotic adherence to the IDSA CAP guideline.

**Methods:**

This single center retrospective cohort study evaluated hospitalized adult patients aged 18 years and older who were started on empiric antibiotics for treatment of CAP in the emergency department. Patients were excluded if they were transferred from an outside hospital or were admitted with COVID-19. Baseline characteristics recorded included categorization of non-severe vs severe pneumonia, risk factors for multi-drug resistant organisms, empiric antibiotic therapy, and culture results. The primary outcome evaluated the percentage of patients who received appropriate empiric antibiotic treatment for CAP according to the IDSA guideline.

**Results:**

A total of 150 patients were included in this study. Approximately 35% of patients presented with severe community acquired pneumonia. The initial antibiotic regimen administered in the emergency department for treatment of suspected CAP was consistent with the IDSA CAP guidelines in 48% of cases. Within twenty-four hours after admission, nearly 25% of cases were optimized to guideline directed therapy. Of the patients with positive culture results, empiric antibiotics were optimized within approximately 48 hours.

**Conclusion:**

More than half of patients who were started on empiric antibiotics for CAP received broad spectrum antibiotics that were not indicated according to the IDSA guidelines. Most patient’s antibiotic regimen was optimized according to the guidelines within 48 hours. Additional stewardship efforts should be implemented in the emergency department to reduce empiric broad spectrum antibiotic use.

**Disclosures:**

**All Authors**: No reported disclosures

